# Decoding Inner Speech Using Electrocorticography: Progress and Challenges Toward a Speech Prosthesis

**DOI:** 10.3389/fnins.2018.00422

**Published:** 2018-06-21

**Authors:** Stephanie Martin, Iñaki Iturrate, José del R. Millán, Robert T. Knight, Brian N. Pasley

**Affiliations:** ^1^Defitech Chair in Brain Machine Interface, Center for Neuroprosthetics, Ecole Polytechnique Fédérale de Lausanne, Lausanne, Switzerland; ^2^Helen Wills Neuroscience Institute, University of California, Berkeley, Berkeley, CA, United States; ^3^Department of Psychology, University of California, Berkeley, Berkeley, CA, United States

**Keywords:** inner speech, electrocorticography, decoding, neuroprosthetics, brain-computer interface

## Abstract

Certain brain disorders resulting from brainstem infarcts, traumatic brain injury, cerebral palsy, stroke, and amyotrophic lateral sclerosis, limit verbal communication despite the patient being fully aware. People that cannot communicate due to neurological disorders would benefit from a system that can infer internal speech directly from brain signals. In this review article, we describe the state of the art in decoding inner speech, ranging from early acoustic sound features, to higher order speech units. We focused on intracranial recordings, as this technique allows monitoring brain activity with high spatial, temporal, and spectral resolution, and therefore is a good candidate to investigate inner speech. Despite intense efforts, investigating how the human cortex encodes inner speech remains an elusive challenge, due to the lack of behavioral and observable measures. We emphasize various challenges commonly encountered when investigating inner speech decoding, and propose potential solutions in order to get closer to a natural speech assistive device.

## Introduction

Neural engineering research has made tremendous advances in decoding motor (Ajiboye et al., 2017) or visual neural signals (Lewis et al., 2015) for assisting and restoring lost functions in patients with disabling neurological conditions. An important extension of these approaches is the development of assistive devices that restore natural communication in patients with intact language systems but limited verbal communication due to neurological disorder. Several brain-computer interfaces have allowed relevant communication applications, such as moving a cursor on the screen (Wolpaw et al., 1991) and spelling letters (Farwell and Donchin, 1988; Gilja et al., 2015; Jarosiewicz et al., 2015; Vansteensel et al., 2016; Pandarinath et al., 2017). Although this type of interface has proven to be useful, patients had to learn to modulate their brain activity in an unnatural and unintuitive way—i.e., performing mental tasks like a rotating cube, mental calculus, movement attempts to operate an interface (Millán et al., 2009), or detecting rapidly presented letters on a screen, such as in the P300-speller (see Fazel-Rezai et al., 2012 for a review) and steady-state visual evoked potentials paradigm(Srinivasan et al., 2006; Nijboer et al., 2008).

As an alternative, people with speech deficits would benefit from a communication system that can directly infer inner speech from brain signals—allowing them to interact more naturally with the world. Inner speech (also called imagined speech, internal speech, covert speech, silent speech, speech imagery, or verbal thoughts) is defined here as the ability to generate internal speech representations, in the absence of any external speech stimulation or self-generated overt speech. While much has been learnt about actual speech perception and production (see Price, 2000; Démonet et al., 2005; Hickok and Poeppel, 2007, for reviews), investigating inner speech has remained a challenging task due to the lack of behavioral output. Indeed, it remains difficult to study this internal neural process due to the difficulty to time-lock precise events (acoustic features, phonemes, words) to neural activity during inner speech. Therefore, substantial efforts have aimed to develop new strategies for analyzing these brain signals.

Investigating the underlying neural representations associated with these different speech features during inner speech is central for engineering speech neuroprosthetic devices. For instance, speech processing includes various processing steps—such as acoustic processing in the early auditory cortex, phonetic, and categorical encoding in posterior areas of the temporal lobe and semantic and higher level of linguistic processes in later stages (Hickok and Poeppel, 2007). One can ask what are the appropriate speech stimulus-neural response mappings to target for efficient decoding and designing optimal communication technologies. For example, a decoding model can target continuous auditory spectrotemporal features predicted from the brain activity. Alternatively, decoding discrete phonemes allows building words and sentences directly.

In this review article, we describe recent research findings on understanding and decoding the neural correlates associated with inner speech, for targeting communication assistive technologies. We focused on studies that have used electrocorticographic (ECoG) recordings in the human cortex, as this promising technique allows monitoring brain activity with high spatial, temporal, and spectral resolution, as compared to electroencephalographic recordings, and the electrodes cover broader brain areas compared to intracortical recordings (Ritaccio et al., 2015). We discuss different decoding and experimental strategies to deal with common challenges that are encountered when tackling inner speech decoding. We consider new avenues and future directions to meet the key scientific and technical challenges in development of a realistic, natural speech decoding device.

In the next section, we first briefly present the properties of electrocorticography, together with its advantages for investigating the neural representation of human speech. We next describe several neuro-computational modeling approaches to neural decoding of speech features.

### Electrocorticographic recordings

**Figure 1:**
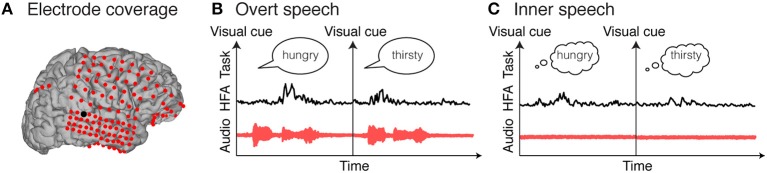
Electrocorticographic recordings. Example of electrocorticographic grid locations overlaid on cortical surface reconstructions of a subject's MRI scan **(A)**. Examples of single trial high frequency activity (HFA) for an electrode highlighted in black in **(A)**. Single trials represent examples of overt speech word repetition **(B)** and inner speech word repetition **(C)**.

Electrocorticography (ECoG), also called intracranial recording or intracranial electroencephalography (iEEG), is used in patients with intractable epilepsy to localize the seizure onset zone, prior to brain tissue ablation. In this procedure, electrode grids, strips or depth electrodes are temporarily implanted onto the cortical surface, either above (epidural) or below (subdural) the dura mater (Figure 1). Because of its invasiveness, only in rare cases, patients are implanted with such electrodes, and it remains exclusively for clinical purposes; nevertheless, the implantation time provides a unique opportunity to investigate human brain functions, with high spatial (millimeters), temporal (milliseconds), and spectral resolution (0–500 Hz). In addition, it covers broad brain areas (typically frontal, temporal, and parietal cortex), which is beneficial given the complex and widely distributed network associated with speech. Finally, electrocorticography is suitable for neuroprosthetic and brain-computer interface applications (Leuthardt et al., 2004, 2006; Felton et al., 2007; Schalk et al., 2007; Blakely et al., 2009; Wang et al., 2013; Kapeller et al., 2014; Moses et al., 2018). Therefore, this technique is an ideal recording candidate for investigating speech functions and for targeting speech neuroprosthetic devices.

ECoG activity contains different signal components (Marshall et al., 2006; Miller et al., 2007; Buzsáki and Wang, 2012; Giraud and Poeppel, 2012) that may relate to different underlying physiological mechanisms, and therefore different mappings between speech stimulus and neural response. For example, the neural representation of speech has been mainly studied using both high frequency (~70–500 Hz) and low frequency (~4–8 Hz).

High frequency activity (HFA; ~70–500 Hz) has been correlated with multiunit spike rate and asynchronous post-synaptic current of the underlying neuronal population (Manning et al., 2009; Whittingstall and Logothetis, 2009; Buzsáki et al., 2012; Lachaux et al., 2012; Rich and Wallis, 2017). In particular, HFA has been shown to robustly encode various speech representations, such as early spectrotemporal acoustic features of speech in the superior temporal gyrus (Pasley et al., 2012; Kubanek et al., 2013)—a region commonly associated with speech perception. In addition, the superior temporal gyrus plays an important role in transforming these acoustic cues into categorical speech units (Chang et al., 2010; Pasley et al., 2011; Mesgarani et al., 2014). HFA in the ventral sensorimotor cortex has been shown to encode acoustic (Pasley and Knight, 2013; Martin et al., 2014; Cheung et al., 2016) and phonetic representations during speech perception, and somatotopically arranged articulator representations (lips, tongue, larynx, and jaw) during speech production (Bouchard et al., 2013; Cheung et al., 2016; Conant et al., 2018).

Low frequencies, such as theta band, have been shown to track the acoustic envelope of speech, to correlate with syllabic rate, and to discriminate spoken sentences (Luo and Poeppel, 2007; Ding and Simon, 2012; Giraud and Poeppel, 2012; Zion Golumbic et al., 2013). In addition, theta rhythms showed significant power changes in Broca's area and temporal language areas during a verb generation task, and showed interactions with high frequency band, through amplitude-amplitude and phase-amplitude coupling (Hermes et al., 2014).

The next section briefly introduces neural decoding models, which have been widely used in the field of speech.

### Decoding models—general framework

Traditionally, cognitive functions have been investigated using a set of stimuli that typically vary along a single dimension of interest (e.g., attended versus not attended target). Brain activity evoked by different stimuli are then averaged and compared in order to provide new insights about the neural mechanisms under study. Conversely, decoding these cognitive functions in real-time for targeting brain-machine interfaces requires more sophisticated predictive modeling. Decoding models allow researchers to apply multivariate neural features to rich, complex and naturalistic stimuli or behavioral conditions (Kay et al., 2008; Kay and Gallant, 2009; Naselaris et al., 2011).

**Figure 2:**
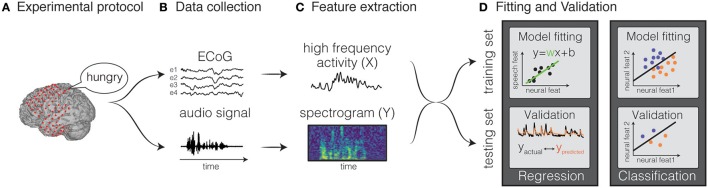
Decoding framework. The general framework for fitting a decoding model is depicted. The first step consists in designing a protocol **(A)** and recording the data **(B)**. Then, input and output features are extracted **(C)**, and the data are split in training and testing set. The training set is used to fit the weights of the model and the testing set is used to validated the model **(D)**. Figures adapted from Holdgraf et al. (2017) with permissions.

A commonly used modeling approach uses a regression framework to link brain activity and a stimulus or mental state representation. For instance, the stimulus features at a given time can be modeled as a weighted sum of the neural activity, as follows:

$$\begin{array}{l} Y\left(t\right)=\;\underset{p}{\sum }w\left(p\right)•X\left(t,p\right) \end{array}$$

where *Y*(*t*) is the stimulus feature at time *t*, *X*(*t, p*) is the neural activity at time *t* and feature *p*, *w*(*p*) is the weight for a given feature *p*. Classification is a type of decoding model in which the neural activity is identified as belonging to a discrete event type from a finite set of choices. Both types of models can use various machine learning algorithms, ranging from simple regression techniques, to more complex non-linear approaches, such as hidden Markov models, support-vector algorithms and neural networks. Holdgraf et al. (2017) provide a review article that illustrates best-practices in conducting these analyses, and included a small sample dataset, along with several scripts in the form of *jupyter* notebooks. The general framework is common to all methods (Figure 2) and consists of the following steps:

1. Feature extraction: input and output features are extracted from the neural activity and from the stimulus features, respectively. Examples of speech representations typically used in decoding models are the auditory frequencies, the modulation rates, or phonemes for natural speech. For neural representations, firing rate from single unit spiking activity, or amplitudes in specific frequency bands are typically extracted from the recorded electrophysiological signal (for example, the high gamma band).
2. Model estimation: models are estimated by mapping input features to output features. The weights are calculated by minimizing a metric of error between the predicted and actual output on a training set. For example, in a linear regression model, the output is a weighted sum of input features.
3. Validation: Once a model is fit, it is then validated on new unseen data not used for training, in order to avoid overfitting and aid generalization to new data. To evaluate the accuracy, the predicted output is compared directly to the original representation.

In the next section, we review ECoG studies that have employed decoding models to understand and decode cognitive states associated with various inner speech representations.

## Decoding inner speech using electrocorticography

A key challenge to understanding the neural representation of inner speech is to quantify the relationship between neural response and the imagined stimulus, from low-level auditory to higher-level speech representations. Several studies have exploited the advantageous properties of intracranial recordings to characterize inner speech representations. For instance, a recent study described the spatiotemporal evolution of high frequency activity during an overt and covert word repetition using trial averaging (Pei et al., 2011b; Leuthardt et al., 2012). In particular, they revealed high frequency changes in the superior temporal lobe and the supramarginal gyrus during covert speech repetition. During a covert verb generation task, high frequency activity (65–95 Hz) showed significant brain activity in Broca's area, in the middle temporal gyrus, and temporal parietal junction, and interacted with theta frequency activity (4–8 Hz) through cross-frequency coupling (Hermes et al., 2014). Finally, a recent study compared the electrocorticographic activity related to overt vs. covert conditions, and revealed a common network of brain regions (Brumberg et al., 2016).

**Figure 3:**
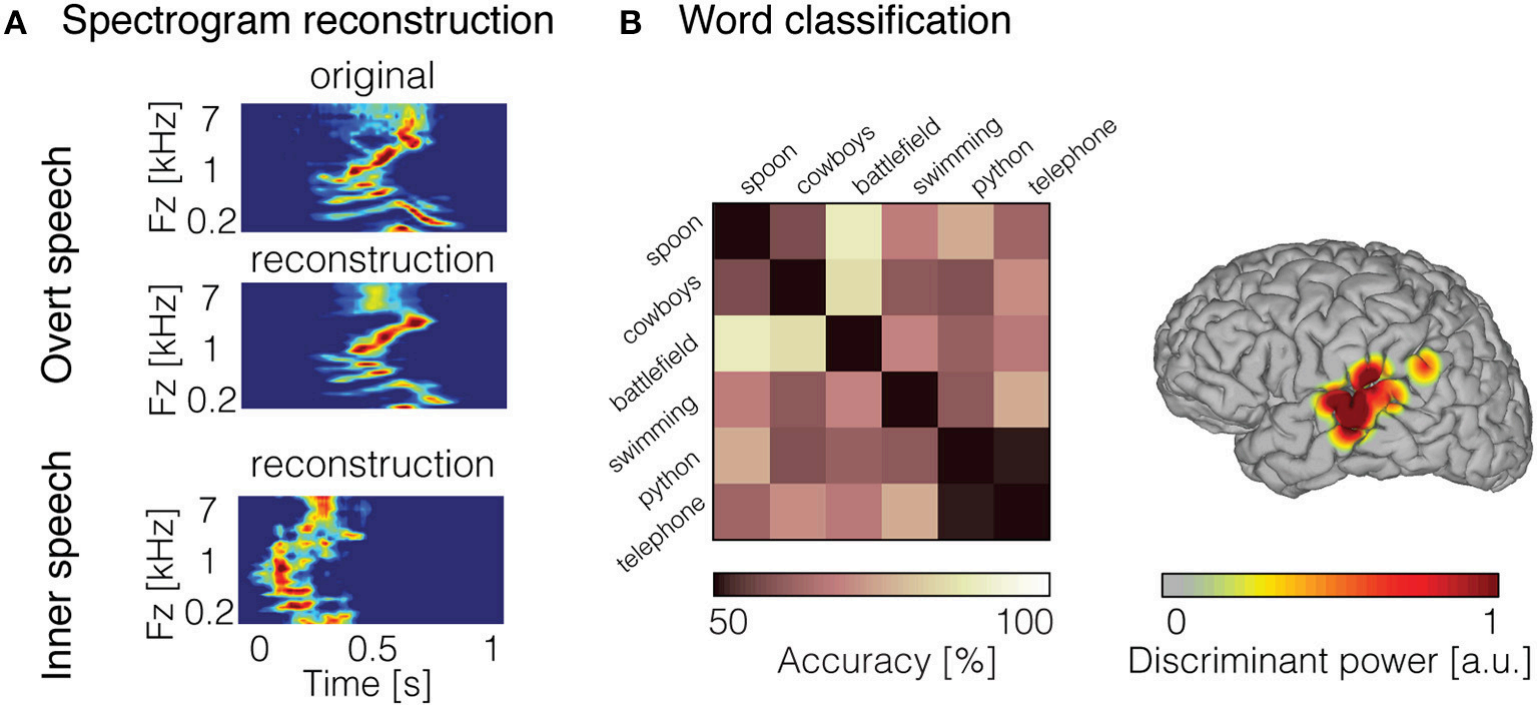
Decoded inner speech representation. **(A)** Examples of overt speech and inner speech spectrogram reconstruction using linear regression models. Original spectrogram of the recorded overt speech sound is displayed (top panel). Reconstruction of the spectrogram for the overt speech condition (middle panel) and inner speech condition (bottom panel). **(B)** Examples of word pair classification during inner speech (left panel). Chance level was 50% (diagonal elements), whereas pairwise classification accuracy (off-diagonal elements) reached 88% and was significantly above chancel level across the 15 pairs of word (mean = 69%). Discriminant information displayed on the surface reconstruction of the participant's brain (right panel) for the classification accuracy shown in the left panel. Figures adapted from Martin et al. (2014, 2016) with permissions.

To directly quantify the relationship between inner speech and neural response, the decoding model framework can be applied. Recently, we used a decoding model approach to reconstruct continuous auditory features from high gamma neural activity (70–150 Hz) recorded during inner speech (Martin et al., 2014). Due to the lack of any measurable behavioral output, standard decoding models (e.g., linear regression) that assume temporal alignment of input and output data are not immediately applicable. One simple approach is to take advantage of prior research demonstrating that speech perception and imagery, to some extent, share common neural mechanisms (Hinke et al., 1993; Yetkin et al., 1995; McGuire et al., 1996; Rosen et al., 2000; Palmer et al., 2001; Aleman, 2004; Aziz-Zadeh et al., 2005; Hubbard, 2010; Geva and Warburton, 2011; Perrone-Bertolotti et al., 2014). Under the assumption that perception and imagery share overlapping neural representations, we built a decoding model from an overt speech condition, and applied this decoder to neural data generated during inner speech. To evaluate performance, the reconstruction in the inner speech condition was compared to the representation of the corresponding original sound spoken out loud—using dynamic time warping (Ellis, 2003)—a temporal realignment algorithm. Results showed that spectrotemporal features of inner speech were decoded with significant predictive accuracy from models built from overt speech data in seven patients (Figure 3A). These findings provided further support that overt and inner speech share underlying neural mechanisms. However, this approach assumes that imagery neural data are generated from a similar neural process as perception. The predictive power of this “cross-condition” model is negatively impacted by discrepancies between perception and imagery neural mechanisms, and is therefore expected to be less optimal compared to directly modeling imagery data in train and test phases.

Beyond relatively low-level acoustic representation, invariant phonetic information is extracted from a highly variable continuous acoustic signal at a mid-level neural representation (Chang et al., 2010). During inner speech, behavioral studies have provided evidence that phoneme substitution errors occurred between phonemes sharing similar features (phonemic similarity effect; Corley et al., 2011), and a similar behavior occurs during overt speech. In addition, brain imaging studies have revealed anatomical brain regions involved in silent articulation, such as the sensorimotor cortex, the inferior frontal gyrus, and temporo-parietal brain areas (Pulvermuller et al., 2006). Recently, electrophysiological studies have shown that the neural activity of a listener that perceives a specific phoneme that has been acoustically degraded, replaced or masked by noise is grounded into acoustic neural representations (Holdgraf et al., 2016; Leonard et al., 2016). This phenomenon, called the phonetic masking effect shows that even in the absence of a given speech sound, the neural patterns correlate with those that would have been elicited by the actual speech sound. These findings suggest that phonemes are represented during inner speech in the human cortex. From a decoding perspective, several studies have succeeded in classifying individual inner speech units into different categories, such as covertly articulated vowels (Ikeda et al., 2014), vowels and consonants during covert word production (Pei et al., 2011a), and intended phonemes (Brumberg et al., 2011). These studies represent a proof of concept for basic decoding of individual speech units, but further research is required to define the ability to decode phonemes during continuous, conversational speech.

While several studies have demonstrated phoneme classification during inner speech (Brumberg et al., 2011; Pei et al., 2011a; Tankus et al., 2012; Ikeda et al., 2014), fewer results are available for word-level classification. Words have been decoded during overt speech from neural signals in the inferior frontal gyrus, superior temporal gyrus, and motor areas (Kellis et al., 2010; Pasley et al., 2012; Martin et al., 2014). In recent work, we classified individual words from high frequency activity recorded during an inner speech word repetition task (Martin et al., 2016). To this end, we took advantage of the high temporal resolution offered by ECoG, and classified neural features in the time domain using a support-vector machine model. In order to account for temporal irregularities across trials, we introduced a non-linear time alignment into the classification framework. Pairwise classification results showed that the classification accuracy was significant across five patients. An example of classification accuracy is depicted in Figure 3B (left panel), where the classification accuracy across the 15 pairs of word were above chance level (average across all pairs = 69%; chance level = 50%). Most of the discriminant information came from the posterior temporal gyrus (Figure 3B; right panel). This study represents a proof of concept for basic decoding of speech imagery, and highlights the potential for targeting a speech prosthesis that allows to communicate a few words that are clinically relevant (e.g., hungry, pain, etc.).

Finally, an alternative study that shows further evidence of acoustic processing during imagery comes from a music imagery study. In this study, we investigated the neural encoding of auditory features during imagery using a novel experimental paradigm that allowed direct modeling of auditory imagery data (as opposed to cross-condition) (Martin et al., 2017). This study is not directly related to speech representations, yet it helps understanding the neural representation of inner subjective experiences, such as general auditory imagery. In addition, evidence has shown that music and speech share common brain networks (Callan et al., 2006; Schön et al., 2010). This study relied on a rare clinical case in which a patient undergoing neurosurgery for epilepsy treatment was also an adept piano player. While previous brain imaging studies have identified anatomical regions active during auditory imagery (Zatorre et al., 1996; Griffiths, 1999; Halpern and Zatorre, 1999; Rauschecker, 2001; Halpern et al., 2004; Kraemer et al., 2005), underlying neural tuning to auditory frequencies in imagined sounds was uncharacterized. ECoG activity was recorded during a task that allowed direct alignment of neural response and the spectrotemporal content of the intended music imagery. The patient played two piano pieces with and without auditory feedback of the sound produced by the electronic piano. The audio signal from the keyboard was recorded in synchrony with the ECoG signal, which allowed synchronizing the audio output with neural activity in both conditions. In this task design, it is assumed that the patient's auditory imagery closely matches the output of the keyboard in both timing and spectral content. This study therefore provided a unique opportunity to apply direct (as opposed to cross-condition) receptive field modeling techniques (Aertsen et al., 1981; Clopton and Backoff, 1991; Theunissen et al., 2000; Chi et al., 2005; Pasley et al., 2012), which describe neural response properties, for example auditory frequency tuning. We found robust similarities between perception and imagery neural representations in both frequency and temporal tuning properties in auditory areas. Furthermore, these findings also demonstrated that decoding models, typically applied in neuroprosthetics for motor and visual restoration, are applicable to auditory imagery, representing an important step toward development of algorithms that could be used in neural interfaces for communication based on auditory or speech imagery.

## Challenges and solutions

An important but challenging step in future research is to describe the extent to which speech representations, such as acoustic processing, phonetic encoding and higher level of linguistic functions apply to inner speech. The lack of behavioral output during imagery and inability to monitor the spectrotemporal structure of inner speech represent a major challenge. Critically, inner speech cannot be directly observed by an experimenter. As a consequence, it is complicated to time-lock brain activity to a measurable stimulus or behavioral state, which precludes the use of standard models that assume synchronized input-output data. In addition, natural speech expression is not just operated under conscious control, but is affected by various factors, including gender, emotional state, tempo, pronunciation, and dialect, resulting in temporal irregularities (stretching/compressing, onset/offset delays) across repetitions. As a result, this leads to problems in exploiting the temporal resolution of electrocorticography to investigate inner speech. In this section, we highlight several additional challenges that are encountered when investigating inner speech, as well as new avenues to improve the decoding outcome.

### Improving task design

The lack of behavioral output and temporal irregularities may be alleviated by designing tasks that maximize the accuracy when labeling the content of inner speech, such as cueing the participants in a rhythmical manner. Despite this, results may still show inconsistencies between the actual cue and the intended speech onset/offset. Alternatively, a verb generation task (Hermes et al., 2014) or picture naming task (Riès et al., 2015) might improve the signal-to-noise ratio, as the cognitive load is more demanding than during a simple word repetition task.

### Training participants

In order to improve accuracy, patients should be familiarized with the tasks before entering in the epilepsy monitoring unit. Indeed, studies have shown that participants with musical training exhibited better pitch and temporal acuity in auditory imagery and enlarged tonotopic maps located in the STG than did participants with little or no musical training (Pantev et al., 1998; Janata and Paroo, 2006; Herholz et al., 2008). As such, we argue it would be beneficial to train subjects on speech imagery, in order to have an increased signal-to-noise ratio and for them to be more consistent in the way of performing the mental imagery. This will improve the performances of any pattern recognition algorithm.

### Finding behavioral markers

Finding a behavioral or neural metric that allows marking more precisely the inner speech time course would reduce temporal variability during inner speech. This will be increasingly important when moving toward asynchronous protocols, i.e., when patients spontaneously produce inner speech, as opposed to experimental protocols that generally employ timing cues. For instance, behavioral and psychology studies rely on indirect measures to infer the existence and properties of the intended inner experience (Hubbard, 2010). For example, participants were instructed to image the pitch of a sine wave tone for a given instrument, and they had to subsequently judge if the timber of a second presented tone matched the timber of the first one (Crowder, 1989). Response times were faster, when the timbre of the second tone matched the timbre of the first one they had to imagine (see Hubbard, 2010 for a complete review). Therefore, objective monitoring of performance and vividness through external markers may allow certain sources of variability during inner speech to be estimated and accounted for in the modeling process.

### Incorporating speech recognition models

Recently, electrophysiological studies on speech decoding have shown promising results by integrating knowledge from the field of speech recognition (Herff et al., 2015; Moses et al., 2016, 2018). Speech recognition has been concerned with the statistical modeling of natural language for many decades, and has faced many problems that are similar to decoding neural pattern associated with speech. As such, we argue that integrating those tools into the field of neuroscience is a necessary element to succeed in the ultimate goal of a clinically reliable speech prosthesis. For instance, speech recognition has developed methodologies that enable the recognition and translation of spoken language into text. This was achieved by incorporating extensive knowledge about how speech is produced and perceived at various phonetic levels (acoustic, auditory, articulatory features), and from advances in computer resources and big data management to build now common applications, such as spellcheck tools, text-to-speech synthesizers, and machine translation programs. Similarly, advanced machine learning models might be more adapted in order to deal with problems associated with speech production temporal irregularities compared to approach like dynamic time warping, which is less robust for noisy data.

### Increasing the amount of data

More complex models with increasing number of parameters can be used, but require more data to train and evaluate the models. When using electrocorticographic recordings, available data are limited. Experimental paradigms usually do not last long to avoid overloading the patients. As an alternative to traditional protocols, researchers are slowly moving toward continuous brain monitoring during the electrode implantation time. This allows increasing the amount of recorded data and is less constraining to the participant as he or she is recorded in the existing hospital environment, e.g., watching television, interacting with relatives and clinicians, reading, etc. Continuous monitoring of speech perception and production may provide sufficient data to develop more complex and robust decoding models.

### Using unsupervised learning

The major problem with recording continuous data is how to label precisely the recordings. Indeed, while it is currently possible to monitor conversations with a microphone, the continuous labeling of categories or events during a movie or a dialogue is a tedious process, and often requires human intervention. In addition, as mentioned earlier, monitoring and labeling internal mental states, such as mood, emotions, internal speech, is problematical. We suggest that unsupervised learning methods might be adapted in this context, and alleviate issues associated with speech segmentation. Unsupervised learning is a type of machine learning algorithms that allows drawing inferences from unlabeled responses, i.e., the labels of the observations are not available. This approach has been used in the field of computer vision, such as to learn the features in order to recognize objects (e.g., a car or a motorcycle).

### Improving the electrode design

Although electrocorticography provides the opportunity to investigate speech neural representation, the configuration, location and duration of implantation are not optimized for experiments, but rather solely for clinical purposes. The design of the intracranial recording electrodes has been shown to be an important factor in motor decoding performance. Namely, the spatial resolution of a cortical surface electrode array depends on the size and spacing of the electrodes, as well as the volume of tissue to which each electrode is sensitive (Wodlinger et al., 2011). Many researchers have attempted to define what the optimal electrode spacing and size could be (Slutzky et al., 2010), but this is still an open area of research. Emerging evidence showed that decoding performance was improved when neural activity was derived from very high-density grids (Blakely et al., 2008; Rouse et al., 2013). However, although a smaller inter-electrode spacing increases the spatial resolution, it poses additional technical issues related to the electrode grid design. Higher density grids placed at specific speech locations would provide higher spatial resolution and potentially enhance the signal's discriminability. Ongoing work in many labs is aimed at increasing the number of recording contacts (Khodagholy et al., 2014) and using biocompatible materials and wireless telemetry for transmission of recordings from multiple electrode implants (Brumberg et al., 2011; Khodagholy et al., 2014). Finally, long-term implantation capability in humans is lacking, as compared to non-human primate studies that showed stable neural decoding for extended periods of time (weeks to months; Ashmore et al., 2012). Reasons for these technical difficulties are the increased impedance leading to loss of signal and increase in the foreign body response to electrodes (Groothuis et al., 2014). Indeed, device material and electrode-architecture influences the tissue reaction. Softer neural implants with shape and elasticity of dura mater increase electrode conductivity and improve the implant-tissue integration (Minev et al., 2015).

## Opportunities

Neural decoding models provide a promising research tool to derive data driven conclusions underlying complex speech representations, and for uncovering the link between inner speech representations and neural responses. Quantitative, model-based characterizations have showed that brain activity is tuned to various levels of speech representation.

The various types of language deficits exemplify the challenge in building a specific speech prosthesis that addresses individual needs. In this regard, the first step is to identify injured neural circuits and brain functions. Once damaged and healthy brain functions are identified, decoding models can be used for the design of effective speech prostheses. In particular, the feasibility to decode various speech representations during inner speech—i.e., acoustic features, phonetic representations, and individual words—suggests that various strategies and designs could be employed and combined for building a natural communication device depending on specific, residual speech functions. Every speech representation has pros and cons for targeting speech devices. For instance, decoding acoustic features opens the door to brain-based speech synthesis, in which audible speech is synthetized directly from decoded neural patterns. This approach has already been demonstrated, where predicted speech was synthesized, and acoustically fed back to the user (Guenther et al., 2009; Brumberg et al., 2010) from intracortical brain activity recorded from the motor cortex. Yet the understandability of the produced speech sounds and the best speech parameters to model remain to be demonstrated. Alternatively, decoding units of speech, such as phonemes or words provides greater naturalness, but the optimal speech unit size to be analyzed, is still a matter of debate—i.e., the longer the unit, the larger the database needed to cover the required domain, while smaller units offer more degrees of freedom, and can build a larger set of complex utterances, as shown in Herff et al. (2015) and Moses et al. (2016). A tradeoff is the decoding of a limited vocabulary of words (Martin et al., 2016), which carry specific semantic information, and would be relevant in a basic clinical setting (“hungry,” “thirsty,” “yes,” “no,” etc.).

An alternative to a speech-interface based solely on brain decoding is to build a system, which acquires sensor data from multiple elements of the human speech production system, and combine the different signals to optimize speech synthesis (see Brumberg et al., 2010, for a review). For instance, recording sensors allow characterizing the vocal tract by measuring its configuration directly or by sounding it acoustically using electromagnetic articulography, ultra-sound, or optical imaging of the tongue and lip. Alternatively, electrical measurements can infer articulation from actuator muscle signals [i.e., using surface electromyography (EMG)] or signals obtained directly from the brain (mainly EEG and ECoG). Using different sensors and different speech representations allow exploiting an individual's residual speech functions to operate the speech synthesis.

Unique opportunities for targeting communication assistive technologies are offered by combining different research fields. Neuroscience reveals which anatomical locations and brain signals should be modeled. Linguistic fields support development of decoding models that incorporate linguistic and contextual specifications—including segmental elements and supra-segmental elements. Combining insights from these research fields with machine learning and speech recognition algorithms is a key element to improve prediction accuracy. Finally, the success of speech neuroprostheses depends on the continuous technological improvements to enhance signal quality and resolution, and allow developing more portable and biocompatible invasive recording devices. Merging various fields together will allow tackling the challenges central to decoding inner speech.

## Conclusion

To conclude, we described the potential of using decoding models to unravel neural mechanisms associated with complex speech functions. Speech representations during inner speech, such as acoustic features, phonetic features and individual words could be decoded from high frequency neural signals. Although, these results reveal a promising avenue for direct decoding of natural speech, they also emphasize that performance is currently insufficient to build a realistic brain-based device. Accordingly, we highlighted numerous challenges that likely precluded better performances, such as the low signal-to-noise-ratio, and the difficulty in monitoring precisely inner speech. As such challenges are solved, decoding speech directly from neural activity opens the door to new communication interfaces that may allow for more natural speech-like communication in patients with severe communication deficits.

## Author contributions

All authors listed have made a substantial, direct and intellectual contribution to the work, and approved it for publication.

### Conflict of interest statement

The authors declare that the research was conducted in the absence of any commercial or financial relationships that could be construed as a potential conflict of interest.
